# Application of clinical nomograms to predicting overall survival and event-free survival in multiple myeloma patients: Visualization tools for prognostic stratification

**DOI:** 10.3389/fpubh.2022.958325

**Published:** 2022-10-17

**Authors:** Jiaxuan Xu, Yifan Zuo, Jingjing Sun, Min Zhou, Xiaoqing Dong, Bing Chen

**Affiliations:** Department of Hematology, The Affiliated Drum Tower Hospital of Nanjing University Medical School, Nanjing, China

**Keywords:** multiple myeloma, prognostic factors, nomogram, risk stratification, survival

## Abstract

**Background:**

This study aimed to develop reliable nomogram-based predictive models that could guide prognostic stratification and individualized treatments in patients with multiple myeloma (MM).

**Methods:**

Clinical information of 560 patients was extracted from the MM dataset of the MicroArray Quality Control (MAQC)-II project. The patients were divided into a development cohort (*n* = 350) and an internal validation cohort (*n* = 210) according to the therapeutic regimens received. Univariate and multivariate Cox regression analyses were performed to identify independent prognostic factors for nomogram construction. Nomogram performance was assessed using concordance indices, the area under the curve, calibration curves, and decision curve analysis. The nomograms were also validated in an external cohort of 56 patients newly diagnosed with MM at Nanjing Drum Tower Hospital from May 2016 to June 2019.

**Results:**

Lactate dehydrogenase (LDH), albumin, and cytogenetic abnormalities were incorporated into the nomogram to predict overall survival (OS), whereas LDH, β2-microglobulin, and cytogenetic abnormalities were incorporated into the nomogram to predict event-free survival (EFS). The nomograms showed good predictive performances in the development, internal validation, and external validation cohorts. Additionally, we observed a superior prognostic predictive ability in nomograms compared to that of the International Staging System. According to the prognostic nomograms, risk stratification was applied to divide the patients into two risk groups. The OS and EFS rates of low-risk patients were significantly better than those of high-risk patients, suggesting a greater function of the nomogram models for risk stratification.

**Conclusion:**

Two simple-to-use prognostic models were established and validated. The proposed nomograms have potential clinical applications in predicting OS and EFS for patients with MM.

## Introduction

Multiple myeloma (MM), the second most commonly diagnosed hematologic malignancy, is characterized by aberrant proliferation of plasma cells in the bone marrow (1, 2). With the aggravation of population aging, the global burden of MM has continued to increase. The incidence of MM has been continuously increasing since 1990, and the incident MM cases have more than doubled worldwide in the past three decades (3, 4). Although the prognosis of MM has improved with the advances in chemotherapy and hematopoietic stem cell transplantation (5), the clinical outcomes vary greatly due to the high heterogeneity of the disease itself, with patient survival ranging from a few months to >10 years (6). Currently, predicting the survival probabilities in MM patients more accurately poses a major challenge.

Staging and typing of MM have always been key to survival prediction and individualized treatment for patients with MM. Owing to the development of detection technology and treatment strategies, the prognostic factors and staging systems for MM are constantly being updated. In 1975, the first MM staging system, the Durie-Salmon (DS) staging system, was proposed (7), which reflects the tumor load and disease progression of MM. The International Staging System (ISS) was first proposed in 2005 (8). It is a simple and effective prognostic stratification method determined by the levels of albumin (ALB) and β2-microglobulin (BMG), and has been widely adopted in disease evaluation. With increasing attention paid to cytogenetic characteristics, the Mayo Stratification of Myeloma and Risk-Adapted Therapy (mSMART) system based on cytogenetic analysis was suggested for the first time in 2007 and updated every few years (9–11). In 2015, the revised ISS (R-ISS) was developed to include cytogenetic abnormalities and lactate dehydrogenase (LDH) levels (12) as a complement to ISS. However, in clinical practice, these staging systems sometimes perform poorly in prognostic assessment, as the survival outcomes may considerably differ in patients with a similar stage owing to the high heterogeneity of MM. Therefore, novel prediction tools and risk stratification systems are urgently required.

The nomogram generates a statistical prediction model by integrating various important biological and clinical factors and creates an individual numerical probability of clinical events, such as death, recurrence, or progression (13). Nomograms are widely used in the field of human health, particularly in cancer prognosis (14, 15). Compared with the traditional tumor clinical staging system, a nomogram not only demonstrates a more effective predictive ability but also allows a faster, more intuitive, and more accurate individual prediction (13, 16, 17). Therefore, nomograms are a promising tool that aids in risk stratification and clinical decision-making for cancer patients. The high heterogeneity of MM makes stratified therapy necessary to help patients achieve optimal efficacy and minimal side effects. In this study, we combined the relevant indicators of ISS and R-ISS staging systems to establish two prognostic nomograms to predict overall survival (OS) and event-free survival (EFS), as a more detailed supplement to the current risk stratification models.

## Materials and methods

### Patient selection

Patient data of the development and internal validation cohorts were extracted from the MM dataset of the MicroArray Quality Control (MAQC)-II study (18, 19). After excluding patients with incomplete laboratory indicators, 350 patients enrolled in total therapy 2 (TT2) were included in the development cohort, and 210 patients enrolled in total therapy 3 (TT3) were assigned to the internal validation cohort. Moreover, we included 56 patients with newly diagnosed MM from May 2016 to June 2019 at Nanjing Drum Tower Hospital as an external validation cohort. All patients with MM were diagnosed according to the diagnostic criteria of the International Myeloma Working Group (20). Follow-up information was obtained from medical records or through telephone interviews. The inclusion criteria were as follows: (a) age >18 years, (b) complete clinical indicators, (c) regular treatment, and (d) complete follow-up information. The study adhered to the principles of the Declaration of Helsinki.

### Data collection

The following demographic and clinical characteristics were collected and analyzed: age at diagnosis, sex, race, immunoglobulin subtype, bone marrow plasma cell (BMPC) percentage, LDH (U/L), ALB (g/dl), BMG (mg/L), hemoglobin (g/dl), creatinine (mg/dl), cytogenetic abnormalities, and ISS stage. The treatment regimens of patients at our center were categorized into two major groups: proteasome inhibitor-based and traditional drug-based. Regarding the primary endpoints, OS was defined as the time from initial MM diagnosis to death from any cause, and EFS was defined as the time from diagnosis to death, relapse, or disease progression.

### Construction and validation of nomograms

Univariate and multivariate Cox regression models were used to determine independent prognostic factors. Two nomogram models were established using independent risk factors to predict the 1-, 3-, and 5-year survival probabilities. The “regplot” package was used to create the dynamic nomograms. The discrimination of the nomograms was evaluated using the concordance index (C-index). Receiver operating characteristic (ROC) curves were also used to assess the discrimination ability of the nomogram models. The predictive performance of nomograms was compared with other models using the area under the curve (AUC) in the development, internal validation, and external validation cohorts. Calibration plots were used to estimate the predictive accuracy of the nomogram models. Calibration curves were generated using 1,000 bootstrap resamples to measure the differences between the actual and predicted survival probabilities. A decision curve analysis (DCA) was performed to determine the potential net benefits of the nomogram models. The clinical usefulness of nomograms was compared with that of the ISS stage through DCA evaluation. Furthermore, based on the median risk score calculated using nomograms, all patients were divided into high- and low-risk groups. The prediction performance of this risk stratification was assessed using Kaplan–Meier curves.

### Statistical analysis

All analyses were implemented using R software (version 4.0.3) in the present study. The normality of continuous variables was tested using the Shapiro–Wilk test. Continuous variables following the normal distribution were expressed as the mean with standard deviation (*SD*); variables that did not follow a normal distribution were presented as medians with interquartile range (IQR), and the Wilcoxon rank-sum test was performed to compare group differences. Categorical variables were examined using the chi-squared test. The log-rank test and Kaplan–Meier curves were used for survival analyses. The median follow-up time was calculated using the reverse Kaplan–Meier method. Hazard ratios (*HRs*) and 95% confidence intervals (*CIs*) were calculated using univariate and multivariate Cox regression analyses. A two-tailed *p*-value of no >0.05 was considered statistically significant.

## Results

### Baseline characteristics of patients with MM

We extracted the data of 560 patients diagnosed with MM from the MAQC-II project and divided the patients into the development and internal validation cohorts according to the treatment regimens received. The demographic and clinical features of 560 patients with MM are presented in Table 1. Another 56 patients treated at our center were recruited as an external validation cohort; their baseline characteristics are summarized in Supplementary Table S1. In these three cohorts, there was a higher percentage of men and patients aged < 65 years. Among the myeloma protein subtypes, IgG had the largest proportion, followed by IgA, free light chain, and others. The proportion of cytogenetic abnormalities and LDH, ALB, and BMG levels were similar in all three cohorts. However, in the external cohort, the proportion of patients with ISS stage II (53.6%) was larger than that of patients with stages I (28.6%) and III (17.9%). In the development and internal validation cohorts, the proportion of stage I patients was greater (52.3%) than that of stage II (26.2%) and III (21.4%) patients. The median follow-up times of patients in the development, internal validation, and external validation cohorts were 70, 43, and 31 months, respectively.

**Table 1:** Baseline clinical and sociodemographic characteristics of 560 MM patients.

**Characteristics**	**All patients**	**Development**	**Internal validation**	***P*-value**
	***N* = 560**	***N* = 350**	***N* = 210**	
**Age (years) (%)**				0.15
< 65	418 (74.6%)	269 (76.9%)	149 (71.0%)	
≥65	142 (25.4%)	81 (23.1%)	61 (29.0%)	
**Sex (%)**				0.08
Female	225 (40.2%)	151 (43.1%)	74 (35.2%)	
Male	335 (59.8%)	199 (56.9%)	136 (64.8%)	
**Race (%)**				1
White	62 (11.1%)	39 (11.1%)	23 (11.0%)	
Other	498 (88.9%)	311 (88.9%)	187 (89.0%)	
**Subtype (%)**				0.01
IgG	312 (55.7%)	194 (55.4%)	118 (56.2%)	
IgA	136 (24.3%)	97 (27.7%)	39 (18.6%)	
FLC	84 (15.0%)	48 (13.7%)	36 (17.1%)	
Other	28 (5.00%)	11 (3.14%)	17 (8.10%)	
BMPC (%) (median [IQR])	45.0 [25.0, 70.0]	50.0 [25.0, 70.0]	42.5 [20.0, 70.0]	0.4
LDH (U/L) (median [IQR])	156 [127, 199]	166 [132, 201]	148 [122, 192]	0.01
ALB (g/dL) (median [IQR])	4.10 [3.70, 4.40]	4.20 [3.73, 4.50]	4.00 [3.70, 4.40]	0.02
BMG (mg/L) (median [IQR])	3.10 [2.10, 4.93]	2.90 [2.00, 4.70]	3.30 [2.30, 5.10]	0.04
Hemoglobin (g/dL) (median [IQR])	11.2 [9.8, 12.6]	11.4 [9.9, 12.7]	11.0 [9.6, 12.3]	0.1
Creatinine (mg/dL) (median [IQR])	1.00 [0.80, 1.22]	1.00 [0.80, 1.30]	1.00 [0.80, 1.20]	0.86
CRP (mg/L) (median [IQR])	4.40 [1.20, 11.0]	4.30 [1.30, 11.80]	4.55 [0.99, 9.00]	0.18
**Cytogenetic abnormalities (%)**				0.65
No	352 (62.9%)	217 (62.0%)	135 (64.3%)	
Yes	208 (37.1%)	133 (38.0%)	75 (35.7%)	
**ISS stage (%)**				0.54
I	293 (52.3%)	189 (54.0%)	104 (49.5%)	
II	147 (26.2%)	87 (24.9%)	60 (28.6%)	
III	120 (21.4%)	74 (21.1%)	46 (21.9%)	

IQR, interquartile range; BMPC, bone marrow plasma cells; LDH, lactate dehydrogenase; ALB, albumin; BMG, β2-microglobulin; CRP, C-reactive protein; ISS, International Staging System. P-value is for comparison between the development and internal validation cohorts.

### Prognostic analyses and nomogram construction

Univariate and multivariate Cox regression analyses were performed to identify independent prognostic indicators of OS (Table 2). Univariate analysis showed that age at diagnosis, BMPC, LDH, ALB, hemoglobin, creatinine, C-reactive protein (CRP), cytogenetic abnormalities, and ISS stage were significantly associated with OS. LDH (*p* = 0.001), ALB (*p* = 0.037), and cytogenetic abnormalities (*p* = 0.001) were identified as independent prognostic factors for OS by multivariate Cox analysis. For the analysis of EFS (Table 3), the following variables were considered significant: BMPC, LDH, BMG, hemoglobin, creatinine, cytogenetic abnormalities, and ISS stage. Only three predictors (LDH, BMG, and cytogenetic abnormalities) remained significant in the multivariate analysis of EFS.

**Table 2:** Univariate and multivariate Cox regression analysis for OS in the development cohort.

**Characteristics**	**Univariate analysis**			**Multivariate analysis**		
	**HR**	**95% CI**	***P-*value**	**HR**	**95% CI**	***P-*value**
Age	1.022	1.004–1.041	0.018	1.013	0.994–1.032	0.174
Sex (male vs. female)	1.131	0.798–1.602	0.489			
BMPC	1.009	1.002–1.015	0.009	0.998	0.991–1.006	0.680
LDH	1.006	1.004–1.008	< 0.001	1.004	1.002–1.006	0.001
ALB	0.577	0.438–0.761	< 0.001	0.722	0.533–0.980	0.037
BMG	1.062	1.043–1.082	< 0.001	1.028	0.987–1.070	0.181
Hemoglobin	0.882	0.806–0.965	0.006	1.013	0.902–1.138	0.827
Creatinine	1.237	1.127–1.358	< 0.001	1.004	0.839–1.201	0.969
CRP	1.012	1.004–1.019	0.004	1.005	0.996–1.014	0.241
Cytogenetic abnormalities (yes vs. no)	2.146	1.524–3.021	< 0.001	1.816	1.259–2.619	0.001
ISS stage (II vs. I)	1.716	1.120–2.630	0.013	1.292	0.777–2.151	0.323
ISS stage (III vs. I)	2.923	1.956–4.370	< 0.001	1.484	0.789–2.790	0.221

OS, overall survival; BMPC, bone marrow plasma cells; LDH, lactate dehydrogenase; ALB, albumin; BMG, β2-microglobulin; CRP, C-reactive protein; ISS, International Staging System.

**Table 3:** Univariate and multivariate Cox regression analysis for EFS in the development cohort.

**Characteristics**	**Univariate analysis**			**Multivariate analysis**		
	**HR**	**95% CI**	***P-*value**	**HR**	**95% CI**	***P-*value**
Age	1.003	0.987–1.021	0.696			
Sex (male vs. female)	1.319	0.937–1.856	0.113			
BMPC	1.009	1.002–1.015	0.007	1.001	0.993–1.009	0.808
LDH	1.003	1.001–1.006	0.013	1.002	1–1.005	0.053
ALB	0.915	0.684–1.223	0.549			
BMG	1.068	1.038–1.100	< 0.001	1.083	1.021–1.15	0.008
Hemoglobin	0.884	0.809–0.967	0.007	0.973	0.87–1.088	0.626
Creatinine	1.216	1.084–1.365	0.001	0.998	0.817–1.219	0.982
CRP	0.988	0.974–1.002	0.083			
Cytogenetic abnormalities (yes vs. no)	1.589	1.139–2.216	0.006	1.657	1.166–2.354	0.005
ISS stage (II vs. I)	1.545	1.037–2.301	0.032	1.147	0.718–1.830	0.567
ISS stage (III vs. I)	2.022	1.347–3.036	0.001	0.875	0.453–1.691	0.691

EFS, event-free survival; BMPC, bone marrow plasma cells; LDH, lactate dehydrogenase; ALB, albumin; BMG, β2-microglobulin; CRP, C-reactive protein; ISS, International Staging System.

**Figure 1:**
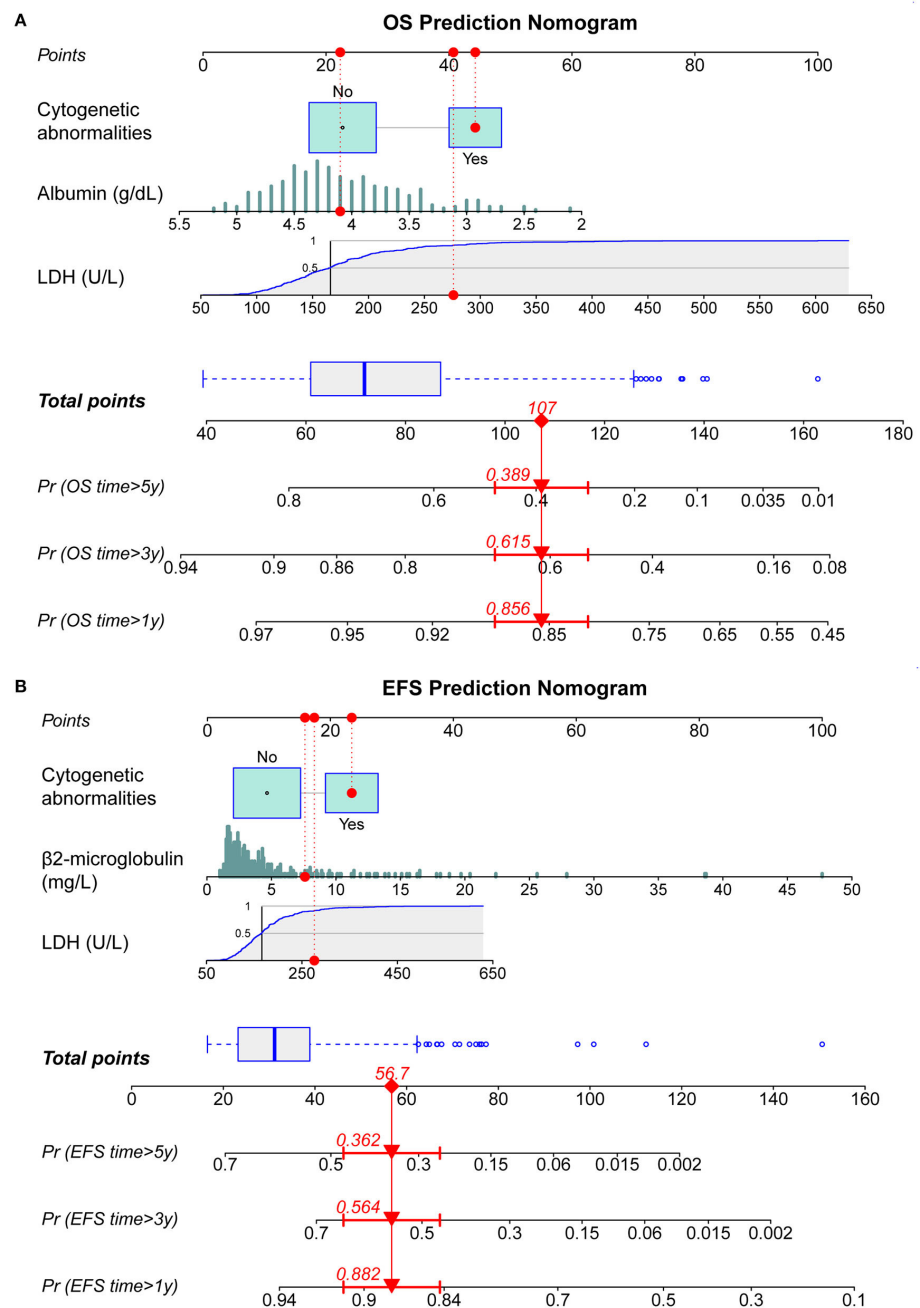
Construction of dynamic nomograms for predicting 1-, 3-, and 5-year survival probabilities. **(A)** Nomogram for predicting overall survival (OS). **(B)** Nomogram for predicting EFS. LDH, lactate dehydrogenase; OS, overall survival; EFS, event-free survival.

By incorporating the independent prognostic factors identified by Cox regression models, we established two predictive nomograms to evaluate the 1-, 3-, and 5-year OS (Figure 1A) and EFS (Figure 1B) rates. Different levels of each risk factor correspond to a particular score, and all scores are summed to obtain a total score plotted on the uppermost total points (TPs) scale. By projecting the total score onto the bottom survival scales, the 1-, 3-, and 5-year OS and EFS rates could be effectively calculated for each MM patient. As shown in Figure 1, a patient with cytogenetic abnormalities, an ALB level of 4.1 g/dl, an LDH level of 276 U/L, and a BMG level of 7.6 mg/L obtained a total score of 107 by using the nomogram for OS prediction, and the 1-, 3-, and 5-year OS probabilities for this patient were 85.6, 61.5, and 38.9%, respectively. Similarly, this patient obtained a total score of 56.7 for EFS prediction and the 1-, 3-, and 5-year EFS rates were 88.2, 56.4, and 36.2%, respectively.

### Evaluation and validation of nomogram performance

The C-index values were used to assess the discrimination of nomograms for survival prediction in the development and validation cohorts (Supplementary Table S2). The nomogram C-index values for predicting both OS and EFS were much higher than the ISS stage C-index values. Good performance of discrimination was confirmed in the internal (*OS*, C-index: 0.749, 95% *CI*: 0.676–0.822; EFS, C-index: 0.732, 95% *CI*: 0.642–0.822) and external (*OS*, C-index: 0.736, 95% *CI*: 0.614–0.858; EFS, C-index: 0.757, 95% *CI*: 0.665–0.849) validation cohorts.

**Figure 2:**
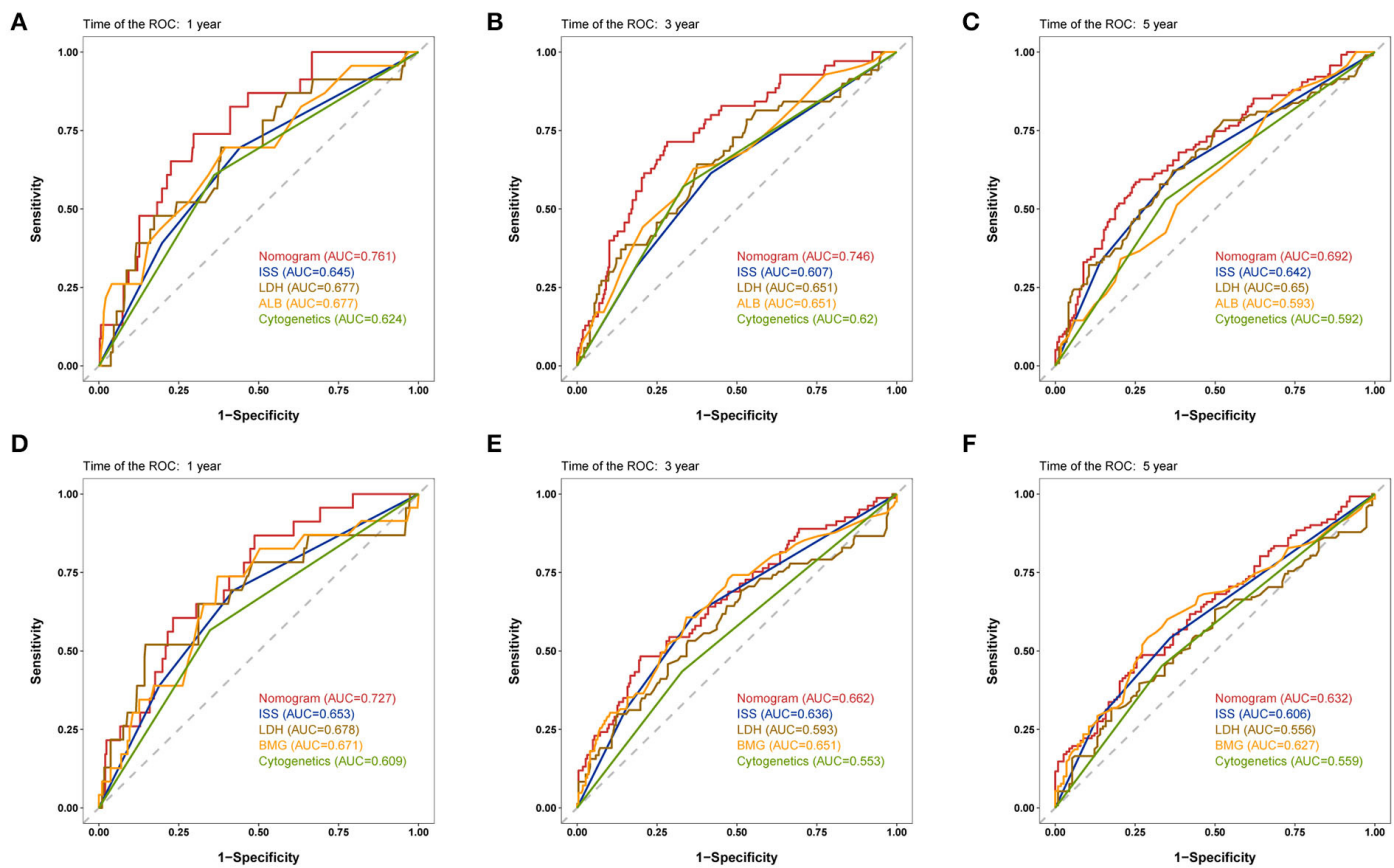
Time-dependent receiver operating characteristic (ROC) curves of the nomogram and other characteristics for prognosis prediction in the development cohort. **(A–C)** 1-, 3-, and 5-year ROC curves for OS prediction. **(D–F)** 1-, 3-, and 5-year ROC curves for event-free survival (EFS) prediction. ROC, receiver operating characteristic; ISS, International Staging System; ALB, albumin; BMG, β2-microglobulin.

The discriminative capability of the two nomograms was examined using ROC curves, and we compared the performance of the nomograms with other characteristics using the AUC values. In the development cohort, the nomogram AUC for 1-year OS prediction was 0.761 (Figure 2A), which was greater than that of the ISS stage, LDH, ALB, and cytogenetic abnormalities (*AUC*: 0.645, 0.677, 0.677, and 0.624, respectively). The AUC values of the nomogram were 0.746 and 0.692 for predicting the 3- (Figure 2B) and 5-year (Figure 2C) OS rates, which were higher than those of the other four prognostic features. For EFS prediction, the AUC of the nomogram for 1-year EFS was 0.727 (Figure 2D), which was higher than that of the ISS stage, LDH, BMG, and cytogenetic abnormalities (*AUC*: 0.653, 0.678, 0.671, and 0.609, respectively). Similarly, the AUC values predicting the 3- and 5-year EFS of the nomogram were greater than those of the other characteristics (Figures 2E,F).

To validate the robustness of the prognostic models, we calculated the AUC values of the nomograms in both the internal and external validation cohorts; the results are shown in Table 4. In the internal validation cohort, the AUC values of the nomogram were 0.804, 0.809, and 0.802 for the 1-, 3-, and 5-year OS, respectively, and 0.818, 0.789, and 0.768 for 1-, 3-, and 5-year EFS, respectively, which are all higher than those of the ISS stage. In the external validation cohort, the nomograms also had superior performance than the ISS stage. These results verified the good predictive performance of the two nomogram models.

**Table 4:** The area under the curve (AUC) values of nomograms and International Staging System (ISS) for overall survival (OS) and event-free survival (EFS) in the internal and external validation cohorts.

**Prognostic models**	**Overall survival**			**Event-free survival**		
	**1-year**	**3-year**	**5-year**	**1-year**	**3-year**	**5-year**
**Internal validation**						
Nomograms	0.804	0.809	0.802	0.818	0.789	0.768
ISS	0.745	0.711	0.679	0.528	0.560	0.621
**External validation**						
Nomograms	0.764	0.776	0.723	0.760	0.772	0.715
ISS	0.621	0.732	0.652	0.655	0.589	0.669

AUC, area under the curve; OS, overall survival; EFS, event-free survival; ISS, International Staging System.

**Figure 3:**
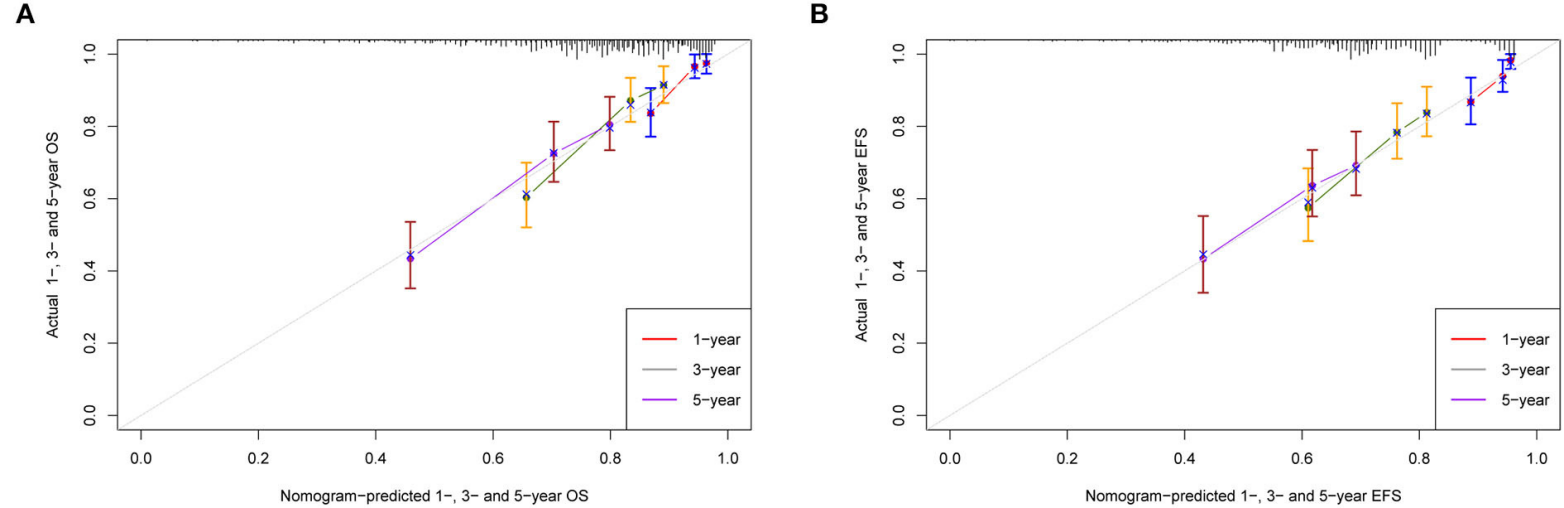
Calibration plots for predicting survival. **(A)** 1-, 3-, and 5-year calibration curves for OS; **(B)** 1-, 3-, and 5-year calibration curves for EFS. OS, overall survival; EFS, event-free survival.

**Figure 4:**
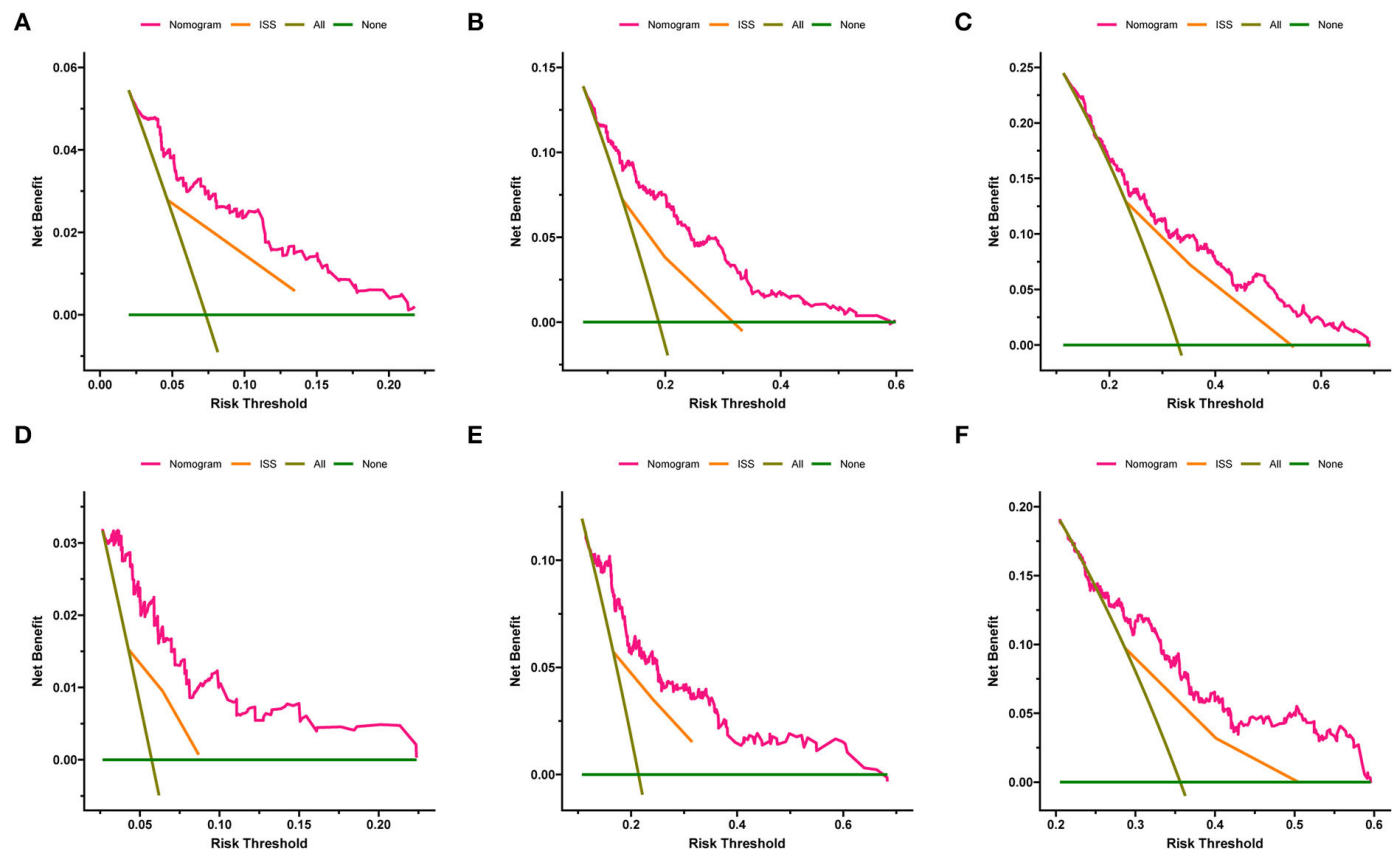
Decision curve analysis of the nomograms and ISS stage in predicting clinical outcomes. **(A)** 1-year OS. **(B)** 3-year OS. **(C)** 5-year OS. **(D)** 1-year EFS. **(E)** 3-year EFS. **(F)** 5-year EFS. OS, overall survival; EFS, event-free survival; ISS, International Staging System.

The calibration curves of 1-, 3-, and 5-year OS and EFS suggested that the actual reference lines showed good agreement with the predicted lines (Figures 3A,B), indicating the reliability of the model predictions. The DCA curves of nomograms for predicting 1-, 3-, and 5-year OS (Figures 4A–C) and EFS (Figures 4D–F) were obtained. The results showed that our predictive nomograms had higher net benefits than the ISS stage, indicating that the nomograms had better clinical implementation significance in predicting both OS and EFS.

### Risk stratification for prognosis

To more accurately predict the clinical outcomes of patients with MM, risk stratification was created based on the TPs of the nomograms. According to the median score (TP: 49) of the nomogram for OS prediction, patients with TP < 49 were classified into the low-risk group, and those with TP ≥ 49 were classified into the high-risk group. In another nomogram for predicting EFS, the median TP was 21. Patients with scores < 21 were assigned to the low-risk group, and those with scores ≥21 were assigned to the high-risk group.

**Figure 5:**
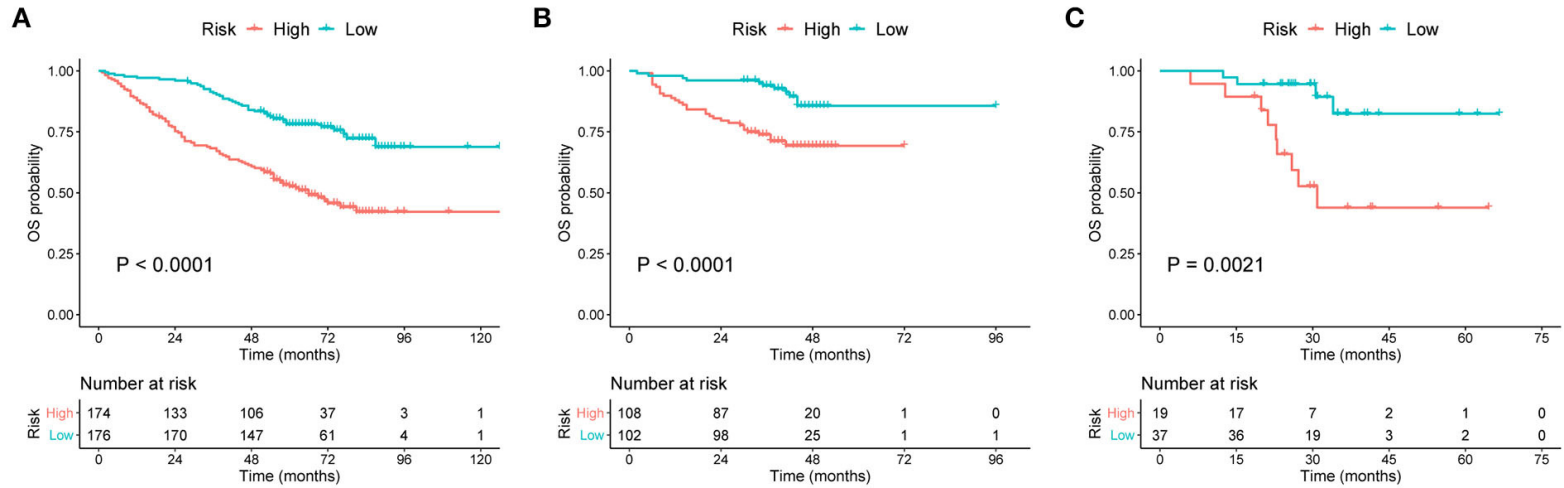
Kaplan–Meier curves of OS for patients grouped by risk levels in the **(A)** development cohort, **(B)** internal validation cohort, and **(C)** external validation cohort. OS, overall survival.

**Figure 6:**
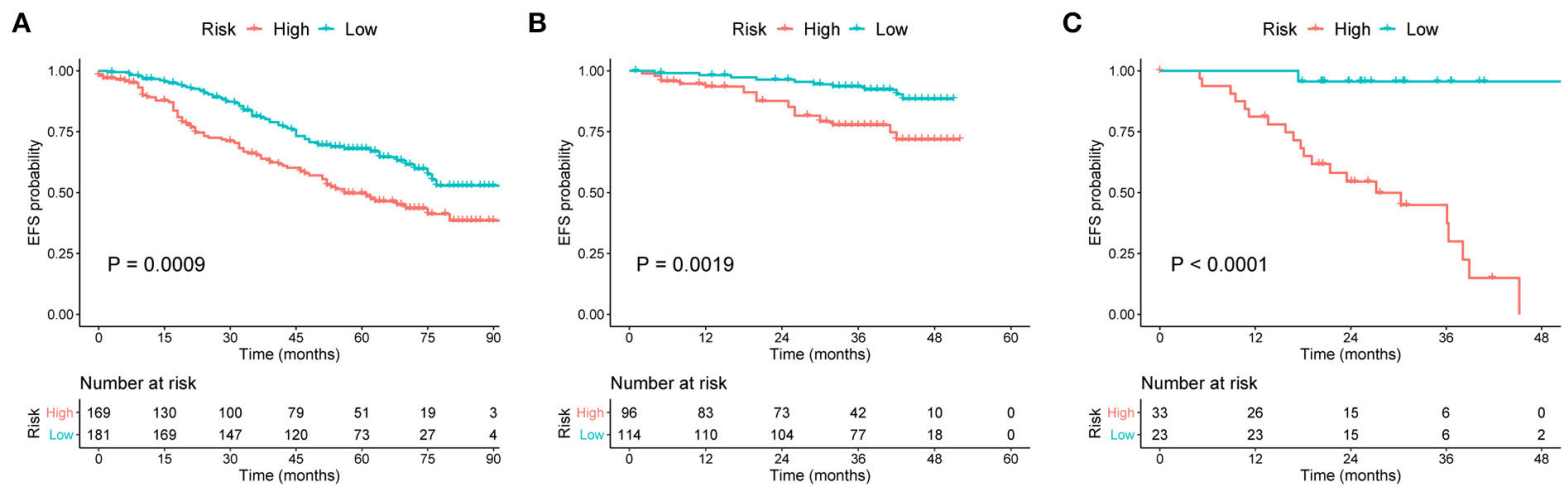
Kaplan–Meier curves of EFS for patients grouped by risk levels in the **(A)** development, **(B)** internal validation, and **(C)** external validation cohorts. EFS, event-free survival.

Survival curves were constructed to assess the risk stratification ability of nomograms. Compared to the low-risk patients, the high-risk patients had significantly poorer OS rates in the development (Figure 5A, *p* < 0.001), internal validation (Figure 5B, *p* < 0.001), and external validation (Figure 5C, *p* = 0.002) cohorts. Likewise, the high-risk group showed significantly worse EFS rates than the low-risk group in the development (Figure 6A, *p* = 0.001), internal validation (Figure 6B, *p* = 0.002), and external validation (Figure 6C, *p* < 0.001) cohorts.

## Discussion

Since the current staging systems for MM cannot consistently provide a precise estimate of the prognosis, new prognostic models for patients with MM need to be developed. Most of the previous nomograms had small samples and lacked external validation, which limits their broader application in predicting clinical outcomes of MM patients. We constructed and validated two nomograms by including 560 American patients from the MAQC-II study and also enrolled Chinese patients for external validation. Our nomograms are the first to be validated in both American and Chinese populations. The nomogram models and risk stratification systems exhibited satisfactory performances in terms of prognosis prediction and risk assessment.

Currently, the ISS is one of the most commonly used staging systems for predicting prognosis in MM, which is based on the measurement of serum ALB and BMG levels (8). The ISS reflects tumor burden at an early disease stage, but the cut-off values of ALB and BMG remain controversial because renal failure could lead to an increase in BMG levels, even in patients with low tumor burden (21). In ISS staging, ALB seems to lose its prognostic significance at a high cut-off level of BMG, whereas it enhances the prognostic value of BMG at a low cut-off level of the latter (22). In our nomograms, we treated ALB and BMG as continuous variables, which helped avoid such controversies of cut-off values and enabled a meticulous quantification of the patient-specific expression levels. Besides, LDH is an important indicator of tumor burden and an easily available blood test; it also has a significant implication on prognosis, even for patients with low or intermediate ISS staging or treated with novel agent-based regimens (23, 24). A previous study has shown that elevated LDH is an adverse prognostic factor independent of the ISS staging for either OS or EFS (25), which is in good agreement with our results. Therefore, we integrated the levels of LDH into the nomograms predicting both OS and EFS. Furthermore, cytogenetic assessment is considered necessary for evaluating MM prognosis (26), and cytogenetic abnormalities were incorporated into the two nomograms to achieve a specific MM staging system.

Herein, we demonstrated the value of ALB, BMG, LDH, and cytogenetic abnormalities in MM prognosis and provided two dynamic nomograms for clinicians or patients to better predict survival outcomes. The constructed nomograms quantified the importance of variables included in the ISS or R-ISS staging systems, with good discrimination and accuracy in both the development and validation cohorts. Once diagnosed with MM, patients can quickly and conveniently predict their clinical outcomes using these common indicators *via* nomogram prediction. Moreover, nomogram-based stratification of prognostic risk could significantly distinguish low- and high-risk patients. Nomograms, along withrisk stratification systems, are expected to function as efficient tools in clinical practice.

Recently, several nomogram models for MM have been proposed. In 2019, Zhang et al. established the first nomogram that predicts the OS in patients with MM. However, the evidence was based on a single-center retrospective study, and the discrimination performance of the nomogram was insufficient (27). Cheng et al. constructed a nomogram for predicting OS and progression-free survival, which included circulating plasma cells as an independent prognostic factor. Another nomogram proposed by Cheng et al. incorporated cytokine MIP-1α as a novel indicator (28, 29). These two nomograms have several common limitations, such as single-center retrospective data, a small sample size, and short follow-up durations. Moreover, they did not include any characteristics associated with cytogenetic abnormalities in their nomogram. Additionally, two other studies in 2021 incorporated the imaging features into the nomograms for OS prediction in MM. Hou et al. adopted chest computed tomography (CT) scans to detect pleural effusion (PE) and constructed a PE-based nomogram to predict survival outcomes for unselected MM patients (30). Li et al. developed a magnetic resonance imaging (MRI)-based radiomics nomogram to predict OS in MM patients (31). Nevertheless, most of the time, the definitive etiology of PE could not be identified due to the low thoracentesis rate. Besides, the assessment of MM-related imaging scores was manually delineated and may be less convenient than other laboratory examinations. Overall, all these nomogram models were based on Chinese populations and lacked external validation.

Our prognostic model has several advantages over the aforementioned nomograms. First, the number of MM patients in the development cohort was relatively large, which guaranteed the reliability of the constructed nomograms. Second, the patients in the training and validation sets were not randomly categorized but were grouped according to their therapy regimens. This could improve the internal validation and increase the applicability of the models for patients receiving different therapies. Third, we focused on the EFS besides OS. The EFS indicator provides a more comprehensive way to assess disease progression or recurrence. Fourth, variables incorporated into the nomograms are easily available, making the predictive nomograms convenient and accessible to use. Lastly, and perhaps most importantly, the patients included for internal validation came from the US, and the patients in the external validation came from China, suggesting that nomogram models are not only applicable to American populations but also to Chinese patients.

Although our nomograms performed well in predicting OS and EFS, the present study has some limitations. First, although a sufficient number of patients was obtained for the development and internal validation cohorts, the sample size for external validation was not relatively small, which may have resulted in limited statistical power. External validations involving more patients and multiple centers are needed to verify the model's performance in the future. Second, owing to the lack of information on high-risk cytogenetics, we could not include the R-ISS staging system or other proposed nomograms to compare with our nomogram models. Besides, we noted that the AUC values for predicting 3- and 5-year EFS in the development cohort were < 0.7, which was rated as acceptable rather than excellent. Further verification is necessary to assess the applicability of the nomogram for predicting long-term survival.

In summary, we confirmed the role of LDH, ALB, BMG, and cytogenetic abnormalities in the clinical course and prognosis of MM. Two nomogram-based prognostic models were developed to predict the OS and EFS rates in patients, which exhibited good predictive performances and were well validated externally. Moreover, the risk stratification systems based on nomograms demonstrated a good degree of discrimination for survival prediction in both the development and validation cohorts, which can contribute to a more precise risk assessment of every MM patient. These simple and easy-to-use visualization tools have the potential to be an effective modality for conducting individualized survival assessments, assisting in risk stratification, and aiding clinical decision-making.

## Data availability statement

The original contributions presented in the study are included in the article/Supplementary material, further inquiries can be directed to the corresponding authors.

## Ethics statement

Written informed consent was obtained from the individual(s) for the publication of any potentially identifiable images or data included in this article.

## Author contributions

All authors listed have made a substantial, direct, and intellectual contribution to the work and approved it for publication.

## Funding

This work was supported by grants from the Jiangsu Provincial Medical Innovation Team (CXTDA2017046) and the Nanjing Medical Science and Technique Development Foundation (YKK20083).

## Conflict of interest

The authors declare that the research was conducted in the absence of any commercial or financial relationships that could be construed as potential conflict of interest.

## Publisher's note

All claims expressed in this article are solely those of the authors and do not necessarily represent those of their affiliated organizations, or those of the publisher, the editors and the reviewers. Any product that may be evaluated in this article, or claim that may be made by its manufacturer, is not guaranteed or endorsed by the publisher.

## References

[1] Kumar SK Rajkumar V Kyle RA Van Duin M Sonneveld P Mateos MV. Multiple myeloma. Nat Rev Dis Primers. (2017) 3:17046. 10.1038/nrdp.2017.46 28726797

[2] Siegel RL Miller KD Fuchs HE Jemal A. Cancer statistics, 2022. CA Cancer J Clin. (2022) 72:7–33. 10.3322/caac.21708 35020204

[3] Cowan AJ Allen C Barac A Basaleem H Bensenor I Curado MP. Global burden of multiple myeloma: a systematic analysis for the global burden of disease study 2016. JAMA Oncol. (2018) 4:1221–7. 10.1001/jamaoncol.2018.2128 29800065 PMC6143021

[4] Zhou L Yu Q Wei G Wang L Huang Y Hu K. Measuring the global, regional, and national burden of multiple myeloma from 1990 to 2019. BMC Cancer. (2021) 21:606. 10.1186/s12885-021-08280-y 34034700 PMC8152089

[5] Pulte D Jansen L Brenner H. Changes in long term survival after diagnosis with common hematologic malignancies in the early 21st century. Blood Cancer J. (2020) 10:56. 10.1038/s41408-020-0323-4 32404891 PMC7221083

[6] Kumar SK Dispenzieri A Lacy MQ Gertz MA Buadi FK Pandey S. Continued improvement in survival in multiple myeloma: changes in early mortality and outcomes in older patients. Leukemia. (2014) 28:1122–8. 10.1038/leu.2013.313 24157580 PMC4000285

[7] Durie BG Salmon SE. A Clinical staging system for multiple myeloma. correlation of measured myeloma cell mass with presenting clinical features, response to treatment, and survival. Cancer. (1975) 36:842–54. 10.1002/1097-0142(197509)36:3&lt;842::AID-CNCR2820360303&gt;3.0.CO;2-U 1182674

[8] Greipp PR San Miguel J Durie BG Crowley JJ Barlogie B Blade J. International staging system for multiple myeloma. J Clin Oncol. (2005) 23:3412–20. 10.1200/JCO.2005.04.242 15809451

[9] Dispenzieri A Rajkumar SV Gertz MA Fonseca R Lacy MQ Bergsagel PL. Treatment of newly diagnosed multiple myeloma based on mayo stratification of myeloma and risk-adapted therapy (Msmart): consensus statement. Mayo Clin Proc. (2007) 82:323–41. 10.4065/82.3.323 17352369

[10] Mikhael JR Dingli D Roy V Reeder CB Buadi FK Hayman SR. Management of newly diagnosed symptomatic multiple myeloma: updated mayo stratification of myeloma and risk-adapted therapy (Msmart) consensus guidelines 2013. Mayo Clin Proc. (2013) 88:360–76. 10.1016/j.mayocp.2013.01.019 23541011

[11] Sonneveld P Avet-Loiseau H Lonial S Usmani S Siegel D Anderson KC. Treatment of multiple myeloma with high-risk cytogenetics: a consensus of the international myeloma working group. Blood. (2016) 127:2955–62. 10.1182/blood-2016-01-631200 27002115 PMC4920674

[12] Palumbo A Avet-Loiseau H Oliva S Lokhorst HM Goldschmidt H Rosinol L. Revised international staging system for multiple myeloma: a report from international myeloma working group. J Clin Oncol. (2015) 33:2863–9. 10.1200/JCO.2015.61.2267 26240224 PMC4846284

[13] Balachandran VP Gonen M Smith JJ DeMatteo RP. nomograms in oncology: more than meets the eye. Lancet Oncol. (2015) 16:e173–80. 10.1016/S1470-2045(14)71116-7 25846097 PMC4465353

[14] Iasonos A Schrag D Raj GV Panageas KS. How to build and interpret a nomogram for cancer prognosis. J Clin Oncol. (2008) 26:1364–70. 10.1200/JCO.2007.12.9791 18323559

[15] Caulfield S Menezes G Marignol L Poole C. Nomograms are key decision-making tools in prostate cancer radiation therapy. Urol Oncol. (2018) 36:283–92. 10.1016/j.urolonc.2018.03.017 29680180

[16] Zhang B Tian J Dong D Gu D Dong Y Zhang L. Radiomics features of multiparametric mri as novel prognostic factors in advanced nasopharyngeal carcinoma. Clin Cancer Res. (2017) 23:4259–69. 10.1158/1078-0432.CCR-16-2910 28280088

[17] Wang S Yang L Ci B Maclean M Gerber DE Xiao G. Development and validation of a nomogram prognostic model for SCLC patients. J Thorac Oncol. (2018) 13:1338–48. 10.1016/j.jtho.2018.05.037 29902534 PMC7678404

[18] Zhan F Huang Y Colla S Stewart JP Hanamura I Gupta S. The molecular classification of multiple myeloma. Blood. (2006) 108:2020–8. 10.1182/blood-2005-11-013458 16728703 PMC1895543

[19] Shi L Campbell G Jones WD Campagne F Wen Z Walker SJ. The microarray quality control (Maqc)-Ii Study of common practices for the development and validation of microarray-based predictive models. Nat Biotechnol. (2010) 28:827–38. 10.1038/nbt.1665 20676074 PMC3315840

[20] Rajkumar SV Dimopoulos MA Palumbo A Blade J Merlini G Mateos MV. International myeloma working group updated criteria for the diagnosis of multiple myeloma. Lancet Oncol. (2014) 15:e538–48. 10.1016/S1470-2045(14)70442-5 25439696

[21] Ooi MG de Mel S Chng WJ. Risk stratification in multiple myeloma. Curr Hematol Malig Rep. (2016) 11:137–47. 10.1007/s11899-016-0307-4 26883334

[22] Mihou D Katodritou E Zervas K. Multiple myeloma staging based on the combination of beta-2-microglobulin and albumin: the role of albumin in the model. Hematology. (2007) 12:527–31. 10.1080/10245330701384161 17852450

[23] Terpos E Katodritou E Roussou M Pouli A Michalis E Delimpasi S. High serum lactate dehydrogenase adds prognostic value to the international myeloma staging system even in the era of novel agents. Eur J Haematol. (2010) 85:114–9. 10.1111/j.1600-0609.2010.01466.x 20477863

[24] Gkotzamanidou M Kastritis E Gavriatopoulou MR Nikitas N Gika D Mparmparousi D. Increased serum lactate dehydrongenase should be included among the variables that define very-high-risk multiple myeloma. Clin Lymphoma Myeloma Leuk. (2011) 11:409–13. 10.1016/j.clml.2011.07.001 21903504

[25] Chim CS Sim J Tam S Tse E Lie AK Kwong YL. Ldh Is an adverse prognostic factor independent of iss in transplant-eligible myeloma patients receiving bortezomib-based induction regimens. Eur J Haematol. (2015) 94:330–5. 10.1111/ejh.12434 25135740

[26] Bataille R Annweiler C Beauchet O. Multiple myeloma international staging system: “staging” or simply “aging” system? Clin Lymphoma Myeloma Leuk. (2013) 13:635–7. 10.1016/j.clml.2013.07.003 24035714

[27] Zhang Y Chen XL Chen WM Zhou HB. Prognostic Nomogram for the overall survival of patients with newly diagnosed multiple myeloma. Biomed Res Int. (2019) 2019:5652935. 10.1155/2019/5652935 31080823 PMC6476154

[28] Cheng Q Cai L Zhang Y Chen L Hu Y Sun C. circulating plasma cells as a biomarker to predict newly diagnosed multiple myeloma prognosis: developing nomogram prognostic models. Front Oncol. (2021) 11:639528. 10.3389/fonc.2021.639528 33747963 PMC7973368

[29] Cheng Q Zhao F Zhang B Zhang Y Cai L Qiao B. Prognostic nomogram incorporating cytokines for overall survival in patients with newly diagnosed multiple myeloma. Int Immunopharmacol. (2021) 99:108016. 10.1016/j.intimp.2021.108016 34385029

[30] Hou ZL Kang Y Yang GZ Wang Z Wang F Yu YX. Pleural effusion-based nomogram to predict outcomes in unselected patients with multiple myeloma: a large single center experience. Ann Hematol. (2021) 100:1789–801. 10.1007/s00277-021-04484-1 33715037

[31] Li Y Liu Y Yin P Hao C Sun C Chen L. Mri-Based Bone marrow radiomics nomogram for prediction of overall survival in patients with multiple myeloma. Front Oncol. (2021) 11:709813. 10.3389/fonc.2021.709813 34926240 PMC8671997

