# Supplementary material for: Application of clinical nomograms to predicting overall survival and event-free survival in multiple myeloma patients: Visualization tools for prognostic stratification
Source: Front Public Health. 2022 Oct 17;10:958325. doi: 10.3389/fpubh.2022.958325 (PMC9618800; doi:10.3389/fpubh.2022.958325)
Supplement: Supplementary file 1 [file Table_1.PDF]

Table S1 Baseline demographic and clinical characteristics in the external validation cohort

**Characteristics**	**Results**
Age (years), n (%)	
<65	35 (62.5%)
⩾65	21 (37.5%)
Sex, n (%)	
Female	23 (41.1%)
Male	33 (58.9%)
Race, n (%)	
White	0 (0 %)
Other	56 (100 %)
Subtype, n (%)	
IgG	27 (48.2%)
IgA	15 (26.8%)
FLC	12 (21.4%)
Other	2 (3.6%)
BMPC (%), median [IQR]	31.8 [19.0, 52.6]
LDH (U/L), median [IQR]	164 [126, 196]
ALB (g/dL), mean (SD)	3.40 (0.68)
BMG (mg/L), median [IQR]	3.54 [2.56, 4.69]
Hemoglobin (g/dL), mean (SD)	8.96 (2.62)
Creatinine (mg/dL), median [IQR]	0.74 [0.66, 0.94]
Cytogenetic abnormalities, n (%)	
No	33 (58.9%)
Yes	23 (41.1%)
ISS stage, n (%)	
I	16 (28.6%)
II	30 (53.6%)
III	10 (17.9%)
Treatment regimens, n (%)	
PIs-based	46 (82.1%)
Traditional drugs-based	10 (17.9%)

IQR, interquartile range; SD, standard deviation; BMPC, bone marrow plasma cells; LDH, lactate dehydrogenase; ALB, albumin; BMG, β2-microglobulin; ISS, International Staging System; PIs, proteasome inhibitors.
