# Supplementary material for: Application of clinical nomograms to predicting overall survival and event-free survival in multiple myeloma patients: Visualization tools for prognostic stratification
Source: Front Public Health. 2022 Oct 17;10:958325. doi: 10.3389/fpubh.2022.958325 (PMC9618800; doi:10.3389/fpubh.2022.958325)
Supplement: Supplementary file 2 [file Table_2.PDF]

Table S2 The C-index values of nomograms and ISS for OS and EFS in the development, internal validation, and external validation cohorts

**Prognostic models**	**Overall survival**		**Event-free survival**	
	**C-index**	**95% CI**	**C-index**	**95% CI**
Development				
Nomograms	0.684	0.639-0.729	0.624	0.575-0.673
ISS	0.615	0.570-0.660	0.591	0.546-0.636
Internal validation				
Nomograms	0.749	0.676-0.822	0.732	0.642-0.822
ISS	0.652	0.572-0.732	0.604	0.508-0.700
External validation				
Nomograms	0.736	0.614-0.858	0.757	0.665-0.849
ISS	0.624	0.463-0.785	0.596	0.473-0.719

C-index, concordance index; CI, confidence interval; OS, overall survival; EFS, event-free survival; ISS, International Staging System.
